# MiR-30c-1-3p targets matrix metalloproteinase 9 involved in the rupture of abdominal aortic aneurysms

**DOI:** 10.1007/s00109-022-02230-2

**Published:** 2022-07-15

**Authors:** Lin Yang, Hong-Gang Sui, Meng-Meng Wang, Jia-Yin Li, Xiao-Feng He, Jing-Yuan Li, Xiao-Zeng Wang

**Affiliations:** 1 College of Medicine and Biological Information Engineering, Northeastern University, Shenyang, Liaoning, 110167 China; 2 Department of Cardiology and Cardiovascular Research Institute, General Hospital of Northern Theater Command, 83 Wenhua Road, Shenyang, 110016 Liaoning China

**Keywords:** Abdominal aortic aneurysm, MicroRNA, MiR-30c-1-3p, Matrix metalloproteinase 9

## Abstract

**Abstract:**

Abdominal aortic aneurysm (AAA) can be fatal if ruptured, but there is no predictive biomarker. Our aim was to evaluate the prognostic potential of microRNAs (miRNAs/miRs) in an AAA mouse model and patients with unruptured AAA (URAAA) and ruptured AAA (RAAA). Among the 64 miRNAs differentially expressed in mice with AAA compared to control, miR-30c-1-3p, miR-432-3p, miR-3154, and miR-379-5p had high homology with human miRNAs. MiR-30c-1-3p plasma levels were significantly lower in patients with RAAA than in those with URAAA or control and tended to negatively correlate with the maximum aortic diameter (*r* =  −0.3153, *P* = 0.06109). MiR-30c-1-3p targeted matrix metalloproteinase (*MMP)-9* mRNA through the coding region and downregulated its expression in vitro. MMP-9 plasma concentrations were significantly higher in the RAAA group than in the URAAA group (*P* < 0.001) and were negatively associated with miR-30c-1-3p levels (*r* =  −0.3671, *P* = 0.01981) and positively–with the maximal aortic diameter (*r* = 0.6251, *P* < 0.0001). The optimal cutoff values for MMP-9 expression and the maximal aortic diameter were 461.08 ng/ml and 55.95 mm, with areas under the curve of 0.816 and 0.844, respectively. Our results indicate that plasma levels of miR-30c-1-3p and MMP-9 may be candidate biomarkers of AAA progression.

**Key messages:**

- Downregulation of miR-30c-1-3p expression and upregulation of its potential target MMP-9 are predictors of the devastation of AAA.
- Downregulation of miR-30c-1-3p expression and its downstream impact on MMP-9 have a potential on predicting the development and rupture of AAA.

**Supplementary Information:**

The online version contains supplementary material available at 10.1007/s00109-022-02230-2.

## Introduction

Abdominal aortic aneurysm (AAA) is caused by degeneration of the arterial wall resulting in continuous dilation of the abdominal aorta, whose diameter can exceed that of the normal aorta by more than 1.5 times. Ruptured AAAs (RAAAs) are associated with a very high overall mortality rate, over 90% [1], and there is an urgent need for reliable biomarkers that can be easily detected in circulation to facilitate systemic screening of the population at risk [2]. However, AAA is a multifactorial condition that could be promoted by genetic and environmental factors, changes in hemodynamics, inflammatory response to pathogenic microorganisms, apoptosis of smooth muscle cells, and inflammation of perivascular adipose tissue. Despite the recent improvements in the diagnostics and therapy of AAA, the associated morbidity and mortality remain high [2, 3]. The molecular mechanisms underlying AAA development are not fully understood and specific biomarkers to predict the initiation and progression of AAA are unknown.

MicroRNAs (miRNAs) are small noncoding RNAs with a length of 18–24 nucleotides, which are known as post-transcriptional regulators of gene expression. MiRNAs bind to complementary sites in 3′-untranslated regions (3′-UTRs) of specific mRNA targets, inhibiting their translation or inducing degradation [4]. A single miRNA can alter the expression of multiple genes and exert effects on several physiological mechanisms. MiRNAs are very stable in the extracellular space and can be found in body fluids such as blood [5]. Many studies have explored the relationship between miRNAs and AAA [6–8] and several thousands of human miRNA sequences with unique expression in different tissues at specific stages of AAA development are expected to serve as new diagnostic biomarkers and therapeutic targets. In this study, we constructed an AAA mouse model under the pathological conditions of both high fat and hypertension, and further adopted miRNA array analysis to determine the differentiated miRNAs that are highly homologous and consistent with AAA diagnosis. We selected relevant genes for verification in patient serum, and examined the correlation between plasma miRNA levels and AAA severity.

## Results

### Evaluation of differential expression of miRNAs in AAA tissues in ApoE-/- mice fed with western diet

**Fig. 1:**
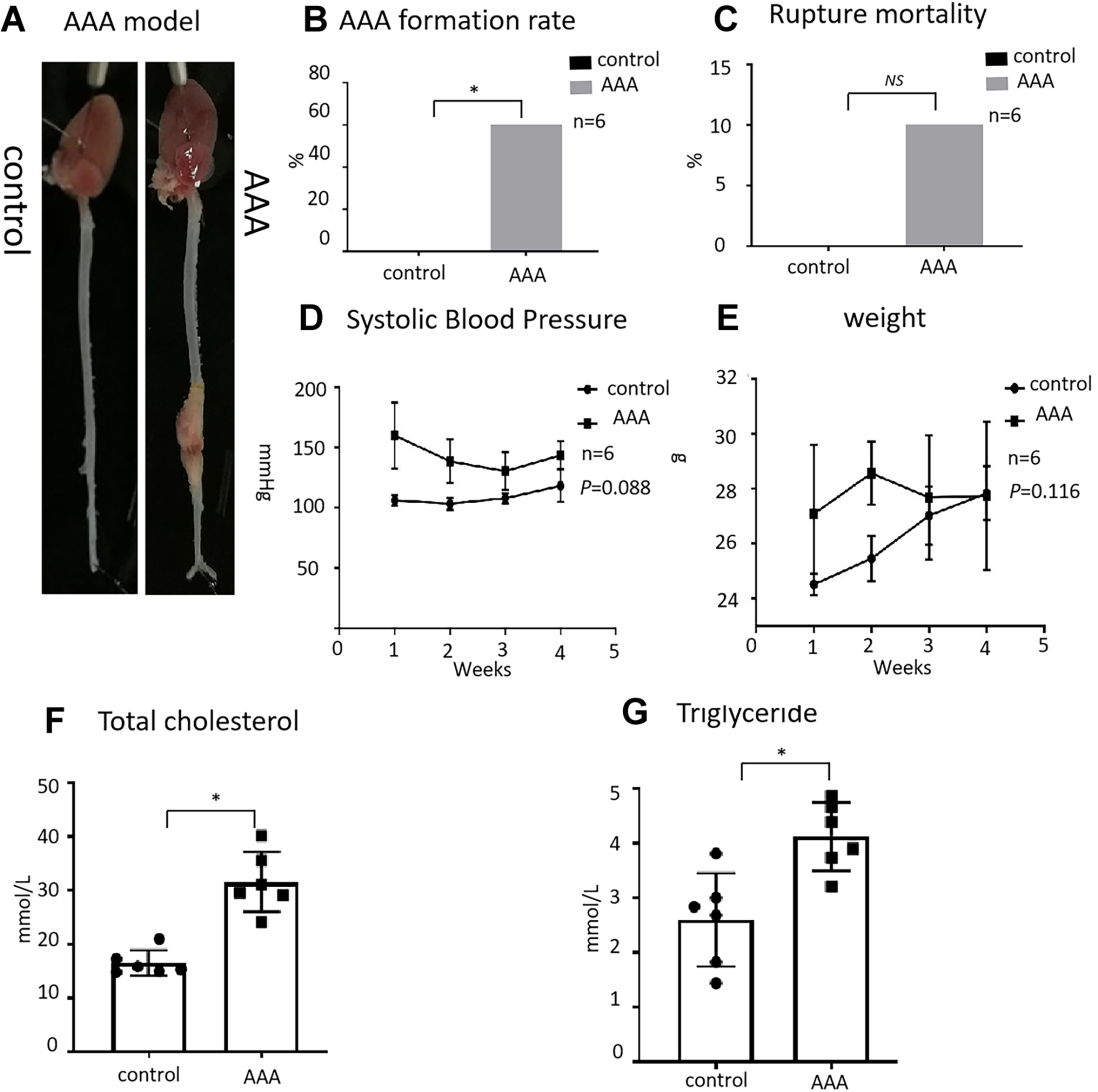
Establishment of the abdominal aortic aneurysm model. **A** Aortic vascularization in a mouse model of angiotensin II stimulation-induced AAA in male ApoE^−/−^ mice. **B**–**C** Comparison of AAA formation rate and rupture mortality between the AAA and control groups. **D**–**E** Comparison of blood pressure and body weight between the AAA and control groups. **F**–**G** Comparison of serum lipid levels between the AAA and control groups. AAA, abdominal aortic aneurysm. Data are expressed as mean ± SEM (*n* = 6). *P < 0.05 AAA vs. control. NS *P* > 0.05 AAA vs. control

**Fig. 2:**
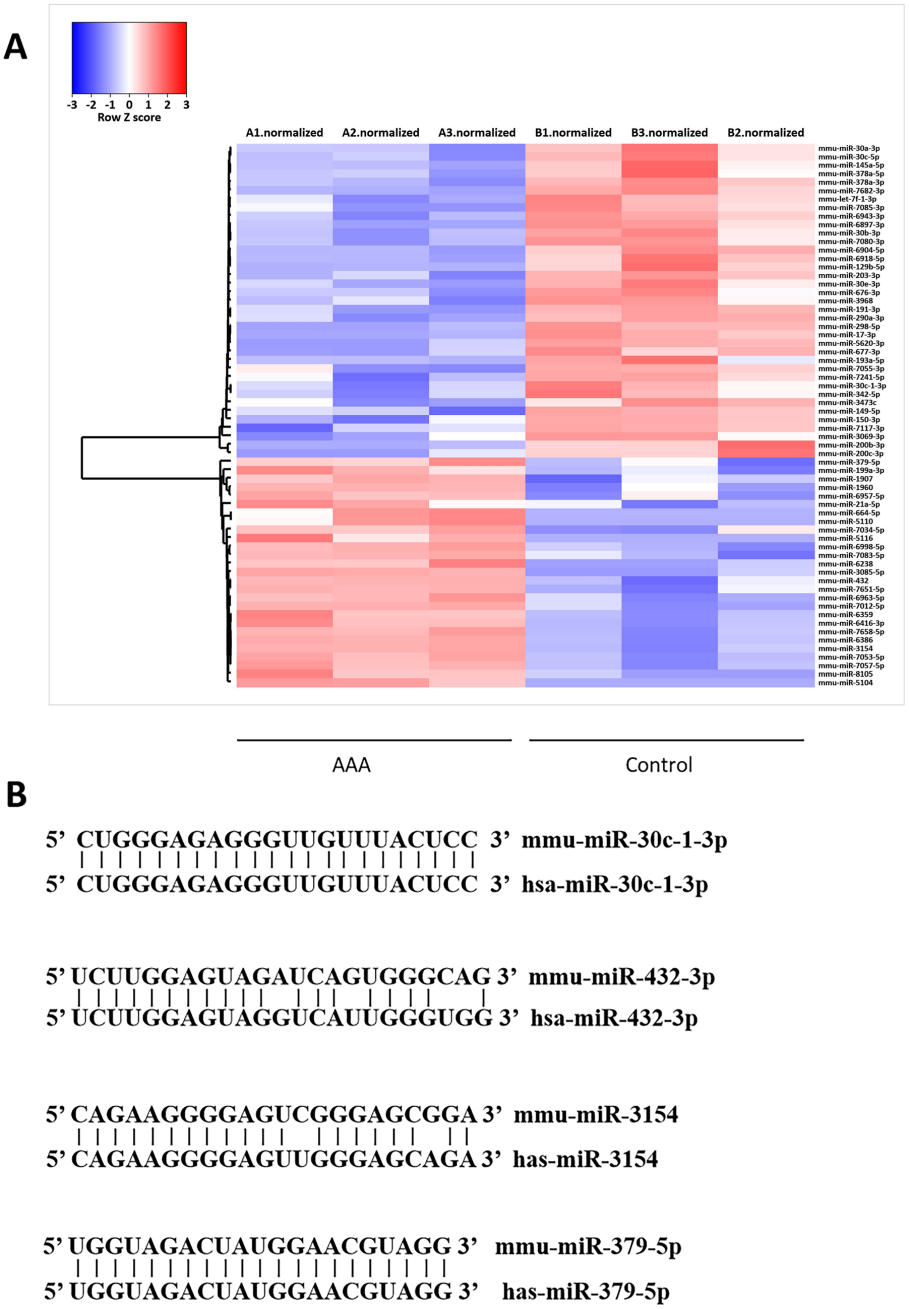
Homology analysis of differential miRNAs in AAA aortic tissue. **A** Heat maps of top of 64 differential miRNA expression in AAA were shown in the dataset. Blue indicates low relative expression, while red indicates high relative expression. **B** miRNAs to homology analysis

To mimic the human AAA injury, an AAA model was created in apolipoprotein E knockout (*ApoE*-/-) mice fed with western diet and infusing angiotensin II (AngII) (1 μg/kg/min) for 4 weeks; the control group was infused with saline and chow diet. The abdominal aorta in AngII-infused mice was more formation and rupture of AAA than in control, indicating successful establishment of the AAA model (Fig. 1A). AAA formation and mortality rates in the AAA group were 60% and 10%, respectively (Fig. 1B, C). Compared to the control group, the average blood pressure was elevated in the AAA group (*P* = 0.088) (Fig. 1D), whereas the body weight slightly reduced (*P* = 0.116) (Fig. 1E). The levels of total cholesterol (TC) (31.60 ± 6.99 *vs.* 17.15 ± 3.53, *P* < 0.01) and triglyceride (TG) (3.96 ± 0.72 *vs.* 2.39 ± 0.79, *P* < 0.01) were significantly higher in the AAA group than in the control group (Fig. 1F–G). Furthermore, miRNA microarray analysis revealed the differential expression of 64 miRNAs in the aorta of AAA mice compared to control mice (Fig. 2A). Among them, we obtained four miRNAs with high homology to human miRNAs for further analysis (Fig. 2B).

### Identification of plasma miRNAs differentially expressed in patients with AAA

Supplemental Table 1 shows clinicopathological characteristics of 20 patients with URAAA, 20 patients with RAAA, and 19 patients with coronary artery disease (CAD) used as a control group. All participants were men, and there were no significant differences in age, diabetes mellitus, hypertension, and smoking habits between the control and AAA (URAAA and RAAA) groups. Statistically significant difference was observed between patients with URAAA and RAAA in the maximum diameter of aortic aneurysms (4.78 ± 0.55 cm *vs*. 6.34 ± 1.33 cm, *P* < 0.001) (Supplemental Table 1).

**Fig. 3:**
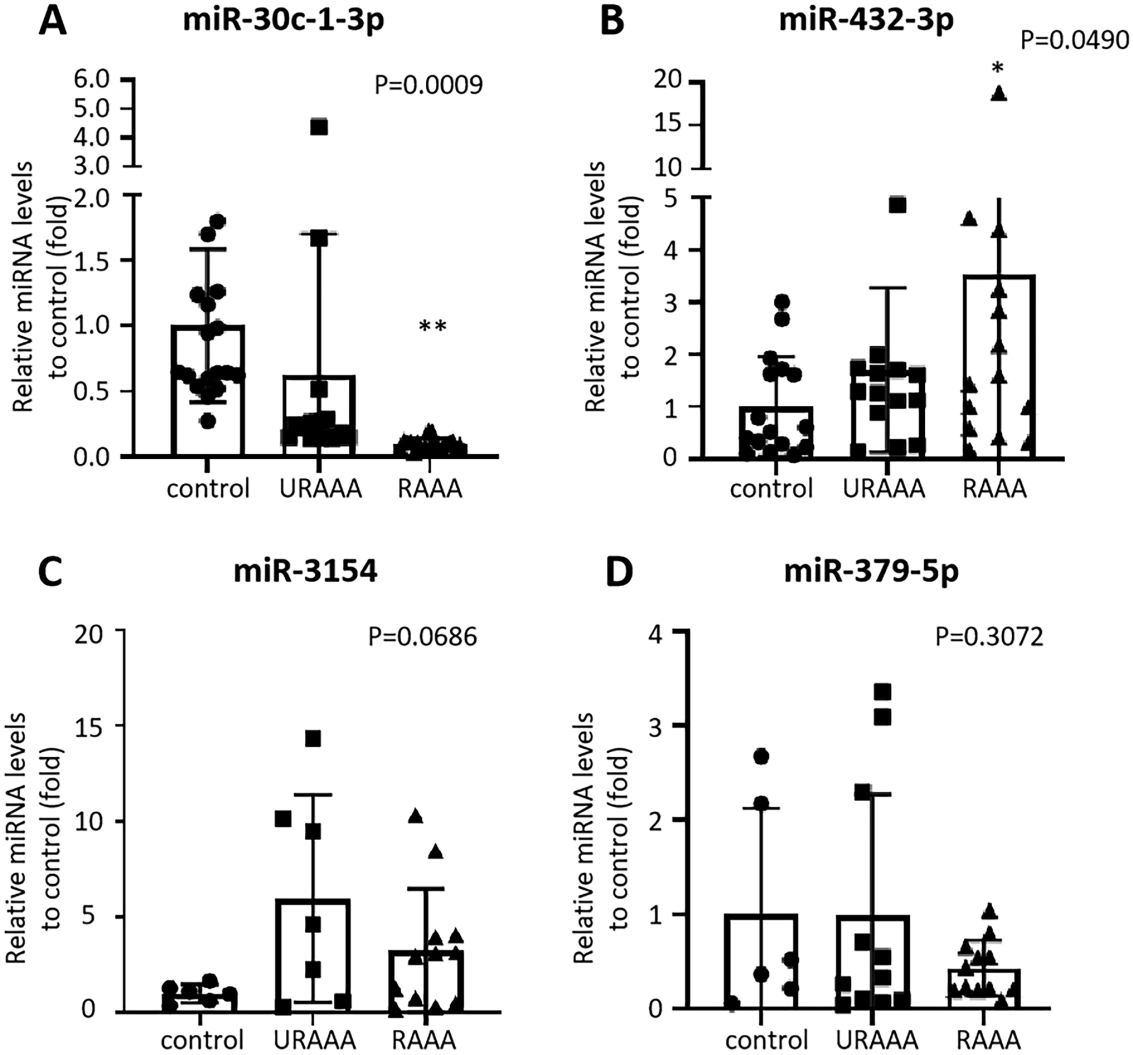
Expression of miRNAs in serum of AAA and control group. We used control miRNA expression as a reference to compare the expression levels of miR-30c-1-3p, miR-432-3p, miR-3154, and miR-379-5p in the plasma of patients with unruptured and ruptured abdominal aortas. Data are represented as mean ± SEM (*n* = 19/20). *p < 0.05 vs. control, **p < 0.01 vs. control

Plasma of patients with RAAA and URAAA and the control group was analyzed four miRNAs expression by quantitative (q)RT-PCR. The results indicated that plasma levels of hsa-miR-30c-1-3p were significantly lower (*P* = 0.0009), whereas those of hsa-miR-432-3p were significantly higher in the RAAA group than in the control and URAAA groups (*P* = 0.0490) (Fig. 3A–B). The expression of miR-3154 was the highest in the URAAA group, followed by the RAAA group and control group and that of miR-379-5p was the lowest in the RAAA group (Fig. 3C–D).

### Correlation between the maximal aortic diameter and miRNA levels

**Fig. 4:**
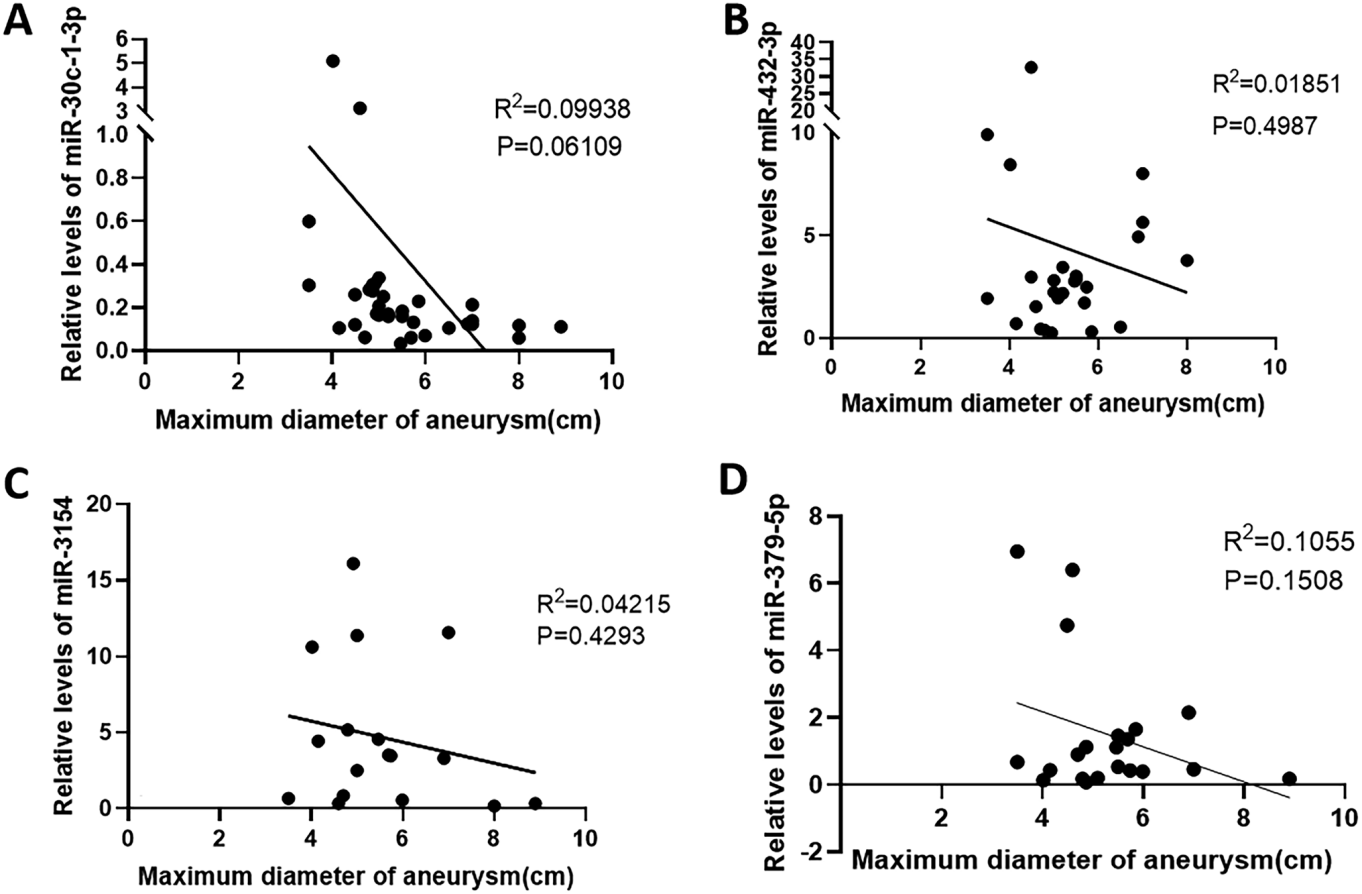
Relationship between the maximum diameter of aorta and the expression of microRNA in serum

Bivariate correlation analysis showed that plasma levels of miR-30c-1-3p in patients with AAA tended to be negatively correlated with the maximal aortic diameter (Pearson correlation coefficient *r* =  −0.3153, *P* = 0.06109) (Fig. 4A). There was no correlation between plasma levels of miR-432-3p (Pearson correlation coefficient *r* = 0.01851, *P* = 0.4987), miR-3154 (Pearson correlation coefficient *r* = 0.04215, *P* = 0.4293), and miR-379-5p (Pearson correlation coefficient *r* = 0.1055, *P* = 0.1508) and the maximal aortic diameter in patients with AAA (Fig. 4B–D).

### Prediction of miRNA target genes and their functional annotation

**Fig. 5:**
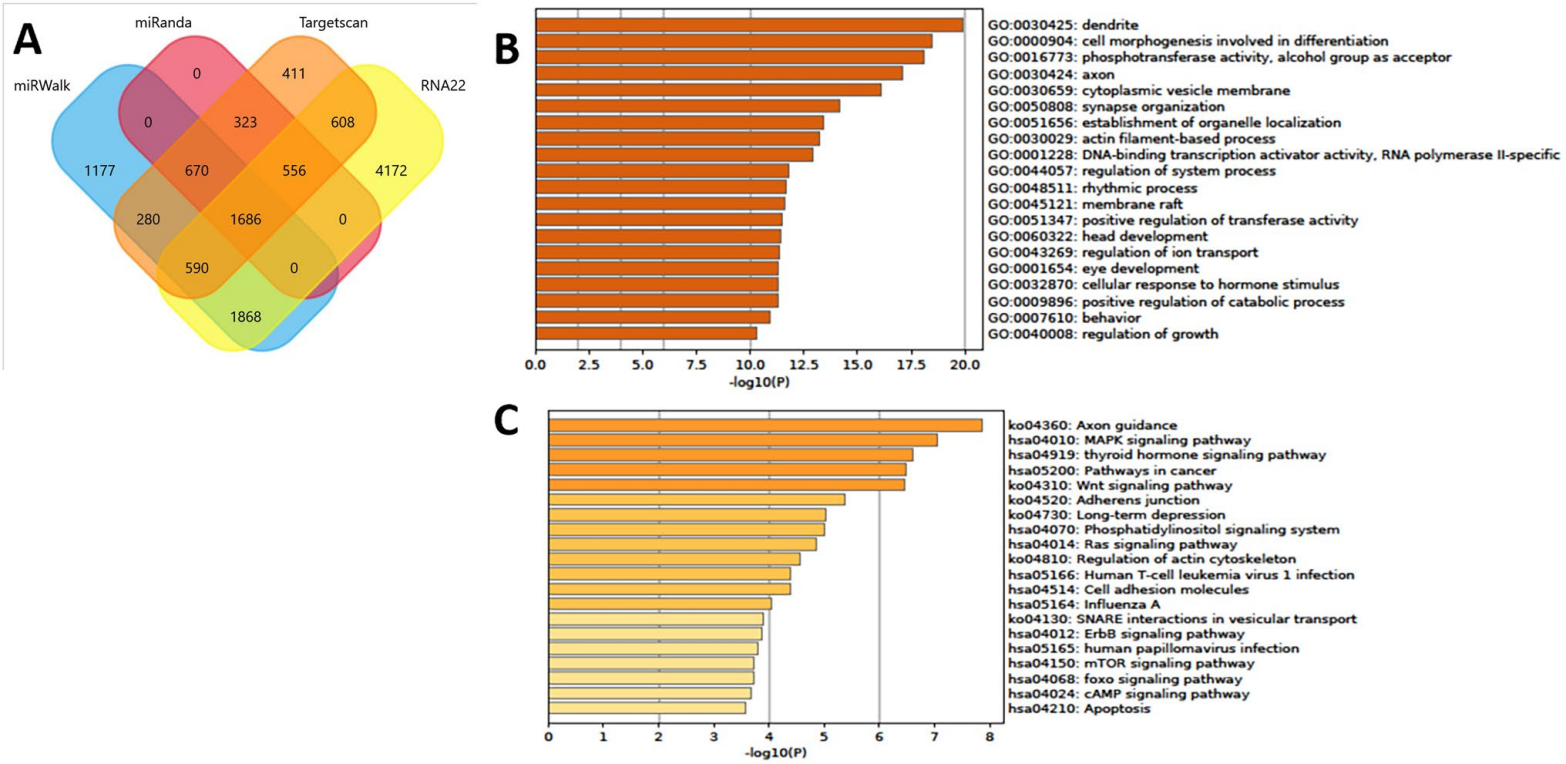
Prediction of miR-30c-1-3p target gene, GO enrichment analysis and the KEGG pathways. **A** Venn diagram showed the collection of miRNA-mRNA relationship in the miRwalk database (blue ellipse), miRanda database(red ellipse), Targetscan database (orange ellipse), and the RNA22 database (yellow ellipse). **B** Gene Ontology enrichment analysis of miR-30c-1-3p. **C** Kyoto Encyclopedia of Genes and Genomes pathways of miR-30c-1-3p

To disclose potential physiological mechanisms regulated by differentially expressed miR-30c-1-3p, its target genes were predicted using miRWalk, miRanda, RNA22, and Targetscan, and the common targets identified by the four databases were revealed by the Venn diagram (Fig. 5A). Next, the target genes were functionally annotated using Gene Ontology (GO) and Kyoto Encyclopedia of Genes and Genomes (KEGG) databases. The target genes of miR-30c-1-3p were distributed according to GO biological processes, cell composition, and molecular function (Fig. 5B). KEGG pathway analysis revealed the top three enriched pathways for miR-30c-1-3p targets, which were axon guidance, MAPK signaling pathway, and thyroid hormone signaling pathway (Fig. 5B).

### Inhibition of MMP-9 expression by miR-30c-1-3p in vitro

**Fig. 6:**
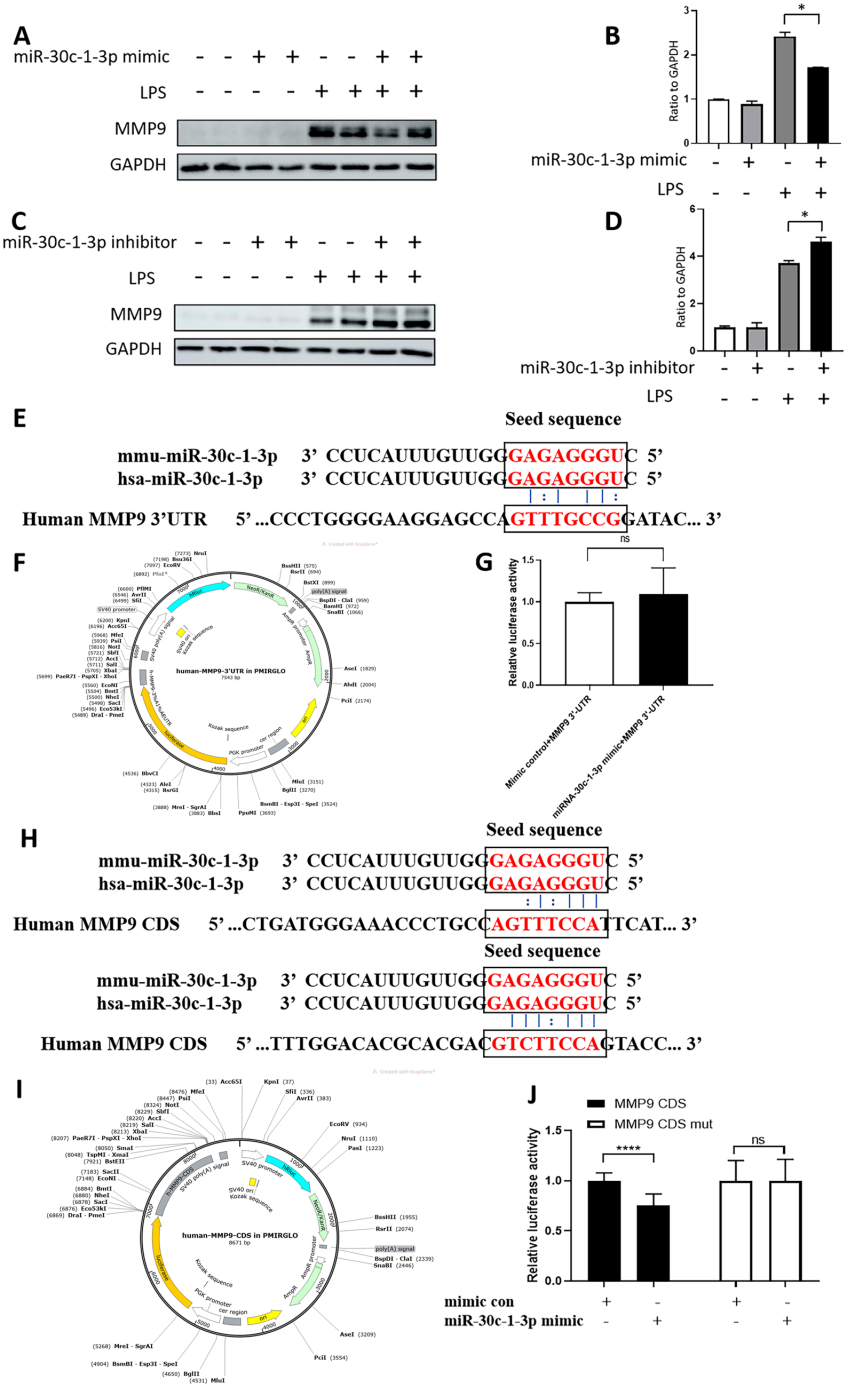
MiR-30c-1-3p overexpression reduces MMP9 expression by targeting its CDS region. **A**–**B** Western blotting and quantification of MMP9 expression in RAW264.7 cells transfected with the mimic control or the miR-30c-1-3p mimic, with or without lipopolysaccharide (LPS) stimulation, *n* = 3. **C**–**D** Western blotting and quantification of MMP9 protein expression in RAW 264.7 cells transfected with the inhibitor control or miR-30c-1-3p inhibitor with or without LPS stimulation, *n* = 3. **E** and **H**, MMP9 sequences targeted by miR-30c-1-3p in humans. **F** and **I**, Reporter vector for the 3′-UTR and CDS region of MMP9. **G** and **J** Luciferase activity in HEK293 cells transfected with MMP9-3′UTR and MMP9-CDS region together with the miR-30c-1-3p mimic and miR-30c-1-3p mimic control, *n* = 6. ns, *P* > 0.05. *, *P* < 0.05. ****, *P* < 0.0001

Prediction analysis identified *MMP-9* as a potential target gene of miR-30c-1-3p. To elucidate whether miR-30c-1-3p directly regulated MMP-9 expression, we induced or reduced miR-30c-1-3p levels in RAW264.7 cells using a miR-30c-1-3p mimic or inhibitor, respectively. Western blot analysis showed that miR-30c-1-3p overexpression decreased the expression of MMP-9 in RAW264.7 cells, whereas its inhibition had the opposite effect (Fig. 6A–B). Using zymography to detect the enzyme activity of MMP9, it was found that the overexpression of miR-30c-1-3p decreased the expression of MMP-9 in RAW264.7 cells supernatant (Supplemental Fig. 1).

### MiR‐30c‐1‐3p inhibited MMP-9 expression through binding to its coding sequence and not to 3′-UTR

To further examine the inhibitory effect of miR-30c-1-3p on MMP-9 expression, we transfected HEK293 cells with a reporter vector for the 3′-UTR of *MMP-9*, which contained miR-30c-1-3p-targeted base pairs (Fig. 6E–F). However, the dual-luciferase reporter assay did not reveal significant differences in luciferase activity between cells co-transfected with 3′-UTR and miR-30c-1-3p mimic or scrambled control (Fig. 6G), indicating that miR-30c-1-3p did not regulate *MMP-9* expression through its 3′-UTR. Interestingly, we found another conserved miR-30c-1-3p target site in the *MMP-9* coding sequence (Fig. 6H–I), and it appeared to be the region through which miR-30c-1-3p regulated *MMP-9* expression according to the reporter assay (Fig. 6J). To validate this result, we mutated the *MMP-9* site targeted by miR-30c-1-3p in the coding sequence and co-transfected HEK293 cells with the mutated vector and miR-30c-1-3p or scrambled control. There was no significant difference in MMP-9 expression between the cells transfected with miR-30c-1-3p or control miR (Fig. 6J), confirming that miR-30c-1-3p inhibited *MMP-9* expression through binding to its coding sequence.

### MMP-9 expression was correlated with miR-30c-1-3p levels and the maximal aortic diameter

**Fig. 7:**
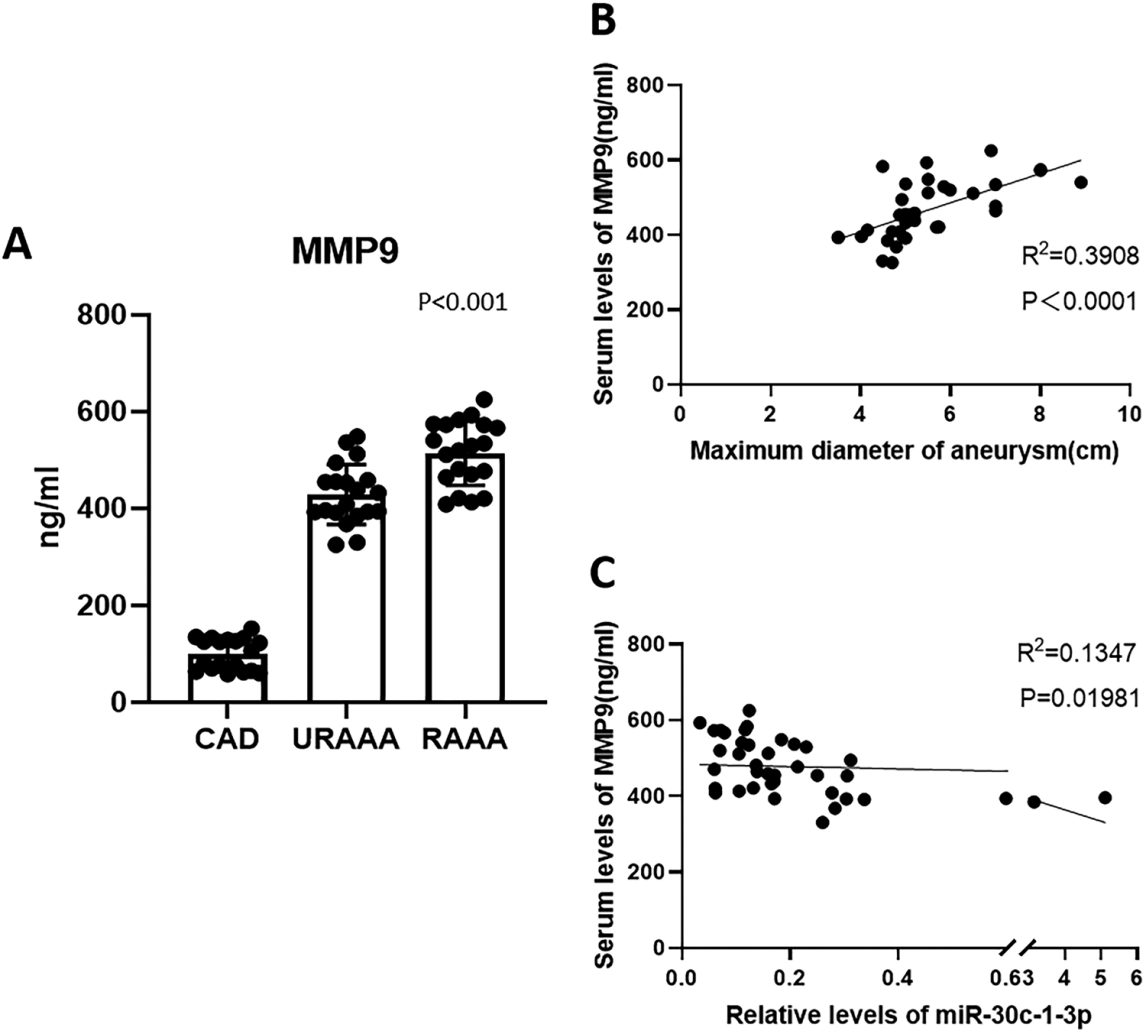
Expression of matrix metalloproteinase 9 and its correlation with aortic maximum diameter and miR-30c-1-3p expression. **A** Expression of matrix metalloproteinase 9 in the plasma of patients with unruptured abdominal aortic aneurysm and ruptured abdominal aortic aneurysm. **B** Relationship between maximal aortic diameter and matrix metalloproteinase 9 expression. **C** Relationship between matrix metalloproteinase 9 and miR-30c-1-3p expression. Data are shown as mean ± SEM (*n* = 19/20)

Analysis of patients’ samples revealed that MMP-9 plasma concentration was increased in the URAAA and RAAA groups compared to control (*P* < 0.001) and in the RAAA group compared to the URAAA group (*P* < 0.001) (Fig. 7A, Supplemental Table 2). Bivariate correlation analysis indicated that MMP-9 levels in plasma of patients with AAA were positively correlated with the maximal aortic diameter (*r* = 0.6251, *P* < 0.0001; Fig. 7B) and negatively with miR-30c-1-3p expression (*r* =  −0.3671, *P* = 0.01981; Fig. 7C).

### MMP-9 expression and the maximal aortic diameter could predict the risk of AAA rupture

**Fig. 8:**
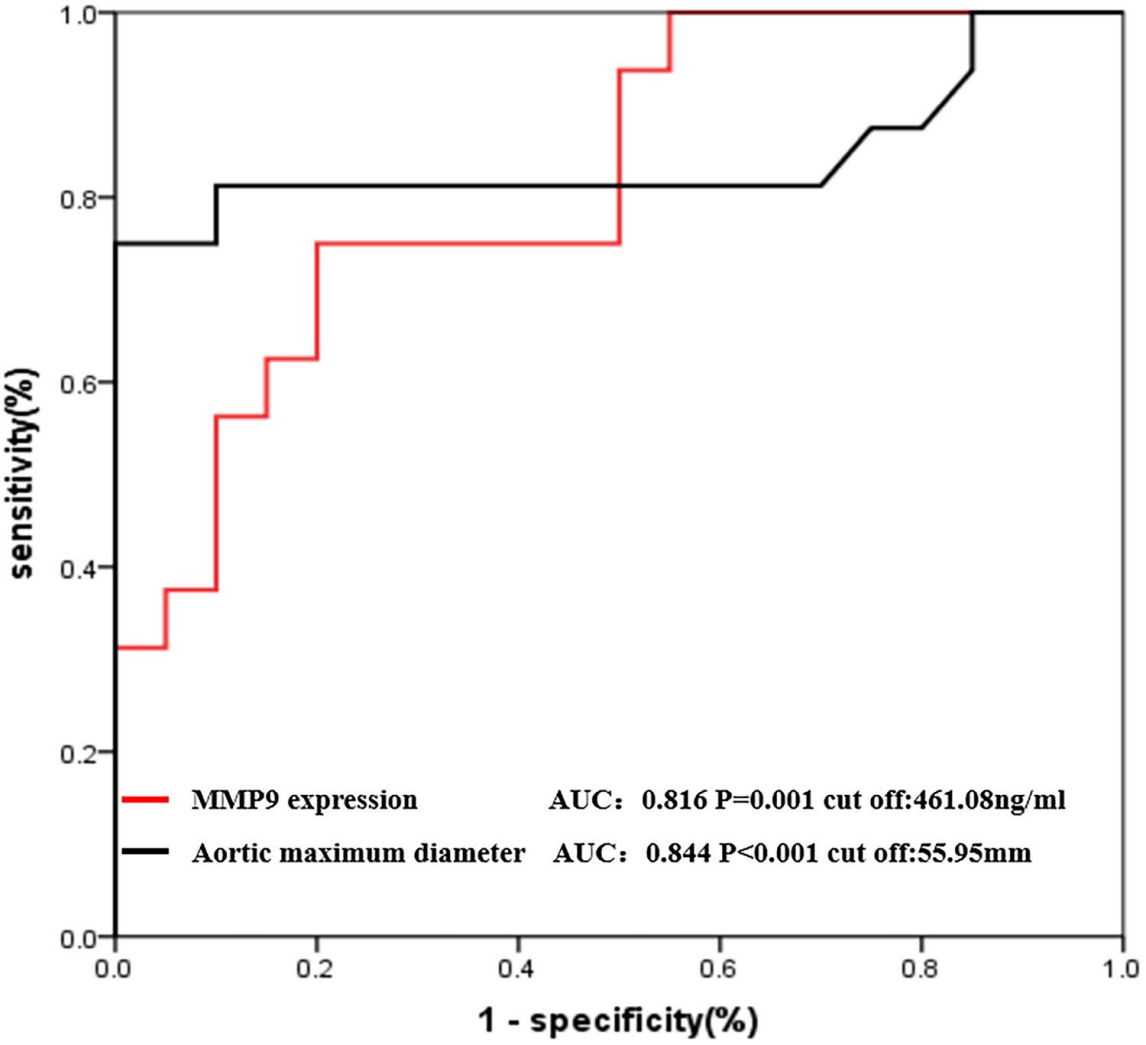
Receiver operating characteristic curve analysis for prediction of ruptured abdominal aortic aneurysm based on the MMP9 expression and aortic maximum diameter

Receiver operating characteristic (ROC) curve analysis demonstrated that MMP-9 expression and the maximum aortic diameter could be used to distinguish patients with URAAA from those with RAAA (Fig. 8). The optimal cutoff values for MMP-9 expression and the maximal aortic diameter were 461.08 ng/ml and 55.95 mm with areas under the curve of 0.816 and 0.844, respectively.

### Agomir-30c-1-3p treatment in vivo

To further assess whether miR-30c-1-3p plays an inhibitory role in AAA formation in vivo, we fed mice a western diet for 8 weeks. We then intermittently injected AgomiR-30c-1-3p and AgomiR-NC through the tail vein for 5 weeks (Supplemental Fig. 2A). The maximal abdominal aortic diameter and AAA formation rate slightly decreased in AgomiR-30c-1-3p-treated mice compared to those in AgomiR-NC-treated mice; however, this difference was not significant (Supplemental Fig. 2B–C).

## Discussion

In the present study, a modified AAA model was used; that is, AAA group mice were fed a western diet for 4 weeks before Ang II injection and continued on the same diet afterwards. The results showed that AAA formation and mortality rates in the AAA group were 60% and 10%, respectively. In previous study, AAA occurred in 33% of mice in the group infused with 1,000 ng/min/kg of Ang II [9]. Similarly, we previously infused Ang II for 4 weeks, using a micro-osmotic pump, in mice fed a chow diet and found that the formation rate of abdominal aortic aneurysms was only approximately 30%. Meanwhile, recent research [10] found that a high-cholesterol diet increases the incidence of AAA in mice, and western diet feeding quickly increases plasma cholesterol concentrations. This further supports our finding that a western diet promotes the occurrence of AAA in mice. Therefore, the modified AAA model fed with western diet in this study can effectively improve the formation rate of AAA, and it is more similar to clinical.

Accumulating evidence indicates that the deregulation of miRNA expression can have profound effects on extracellular matrix remodeling, cell cycle, aging, and inflammation [7] and is associated with many diseases, including cancer, cardiovascular diseases, and neurological disorders [11–13]. MiRNAs are distinguished by high extracellular stability and can be detected in blood and other body fluids; therefore, circulating miRNAs share many characteristics essential for biomarkers, such as sensitivity, specificity, and a long half-life in the sample, as well as rapid and cost-effective laboratory detection.

In this study, we showed that the miRNA expression profile in plasma of patients with AAA differed from that of patients with CAD and that the levels of circulating miR-30c-1-3p was different in patients with URAAA and RAAA, suggesting that these miRNAs could be novel candidate biomarkers for AAA progression. Among the four identified miRNAs, miR-30c-1-3p plasma levels were significantly lower in patients with RAAA than in those with URAA. Furthermore, there was no previous study on miR-30c-1-3p related to AAA. In search for miR-30c-1-3p targets, we identified MMP-9, which showed negative correlation with miR-30c-1-3p levels and positive correlation with the maximal aortic diameter. According to ROC curves for RAAA prediction, MMP-9 expression or the maximal aortic diameter was greater than 461.08 ng/ml or 55.95 mm may indicate the risk of AAA rupture, respectively. These results implicate the miR-30c-1-3p/MMP-9 axis in AAA progression to rupture.

It has been shown that miRNA-30c attenuates hyperlipidemia, hypercholesterolemia, and atherosclerosis in mice [14, 15] and plays an important role in angiogenesis [16]. Our KEGG pathway analysis revealed that miR-30c-1-3p targets were enriched in the axonal guidance, thyroid hormone signaling, and MAPK signaling pathways. Indeed, previous studies indicate that the MAPK pathway is the key biochemical mechanism involved in AAA [17]. Furthermore, MMP-9, one of the main miR-30c-1-3p targets, has been reported to contribute to the rupture of AAAs [18], whereas MMP-9 plasma concentrations are positively correlated with the AAA growth rate [19] and AAA rupture [20, 21]. These findings are consistent with our results that MMP-9 levels in patients with RAAA were significantly higher than those in patients with URAAA and that MMP-9 concentration exceeding 461.08 ng/ml may indicate the risk of AAA rupture.

As a rule, miRNAs bind to complementary sites in 3′-UTRs of specific mRNA targets [22] and through this, exert their effects on diverse cellular processes, including cell proliferation, differentiation, and apoptosis [23, 24]. An interesting finding of our study is that miR-30c-1-3p regulated MMP-9 expression through binding to its coding region rather than 3′-UTR, which is, to the best of our knowledge, shown for the first time.

Finally, in the in vivo study, miR-30c-1-3p overexpression slightly reduced the maximal abdominal aortic diameter and AAA formation rate in an Ang II-infused mouse model using AgomiR-30c-1-3p. However, the difference was not statistically significant. The reason might be attributed to (1) a relatively small number of mice in each group and/or (2) an insufficient dose of AgomiR-30c-1-3p. We will, therefore, use a AAA mouse model with a global mir-30–1-3p knockout to determine its role in AAA development in future studies.

In conclusion, our results suggest that the downregulation of miR-30c-1-3p expression can result in the upregulation of its potential target MMP-9, increase in the aorta diameter, and acceleration of AAA development. A change in miR-30c-1-3p serum levels may indicate the stage of AAA progression and serve as a potential biomarker of AAA development and the risk of AAA rupture.

## Methods

### Ethics

Both human serum samples and animal studies were approved by the Clinical Research Committee and the Institutional Animal Care and Use Committee of General Hospital of Northern Theater Command respectively. All human studies adhered to the guidelines of the Declaration of Helsinki. All participants in the experiment provided informed consent. All animal experiments complied with the Guide for the Care and Use of Laboratory Animals published by the United States National Institutes of Health.

### Animal model and samples

We used 6–8-week-old male *ApoE*-/- mice (weight 20–25 g) purchased from Nanjing Model Animal Center (Nanjing, China). Mice were infused with Ang II (ApexBio Technology, Houston, USA) at 1 μg/kg/min (the dose was based on a previous study [9]) for 4 weeks using a micro-osmotic pump (model 1004, ALZET Osmotic Pumps, Cupertino, CA, USA). AAA group mice were fed a western diet for 4 weeks before Ang II injection and continued on the same diet afterwards. Control group mice were fed a chow diet and infused with saline. The level of abdominal aorta from the left renal artery to the suprarenal area was analyzed by echocardiography. Images of the whole aorta were obtained by scanning the long and short aorta axes. Each mouse was scanned three times by an investigator blinded to the nature of treatment. Blood pressure was measured by the tail-cuff method using a Softron BP2010A Blood Pressure Meter (Softron Beijing Biotechnology Co., Ltd, Beijing, China). The tail was fixed to the sphygmomanometer, and sensors were used to record blood pressure indirectly. Blood pressure was measured at least three times in each mouse at 0, 7, 14, and 28 days. Mice that died during the experiment were dissected, and RAAAs were detected based on dilated perivascular blood clots or abdominal bleeding; these mice were included in the assessment of the tumor formation rate and risk of rupture. The surviving mice were euthanized under anesthesia at day 28, and the aorta (from the ascending aorta to the iliac artery) was dissected out. The AAA specimens were frozen at −80 ℃, transferred into centrifuge tubes, sealed on dry ice, and mailed to BioMiao Biological Technology Co., Ltd. (Beijing, China) for microarray analysis.

For in vivo validation experiments, the mice were fed a western diet for 3 weeks and then randomly divided into two groups (*n* = 7–8 per group). Animals were administered agomiR-30c-1-3p (20 nmol/mouse, RiboBio, Guangzhou, China) or agomiR-NC (20 nmol/mouse, RiboBio, Guangzhou, China) through the tail vein twice 1 week. Afterwards, animals was administered agomiR-30c-1-3p or agomiR-NC through the tail vein twice a week, meanwhile infused with AngII using a micro-osmotic pump and fed a western diet for 4 weeks. AgomiR-30c-1-3p is an analog of miR-30c-1-3p, which can increase the expression of miR-30c-1-3p in mice; agomiR-NC was used as the negative control.

### Microarray analysis

miRNA expression in mouse aortas was analyzed by BioMiao Biological Technology Co., Ltd (Beijing, China) using the Agilent Mouse miRNA Microarray Kit, Release 21.0, 8 × 60 K (Design ID:070,155; Agilent Technologies, Santa Clara, CA, USA), which contained 1902 probes for mature miRNA.

### Patient cohorts

In total, 59 patients were included in the study: 20 with RAAA, 20 with URAAA, and 19 with CAD (control group). To assess the maximal aortic diameter and the presence of an entry tear, all patients underwent computed tomographic angiography (CTA) during hospitalization, as previously described [25]. CTA was carried out using a Siemens Somatom Sensation 64 CT Scanner after administration of intravenous boluses containing 80–150 mL of nonionic contrast medium; three measurements were performed and the mean values were calculated.

### miRNA isolation from blood samples and qRT-PCR

Peripheral venous blood was collected from patients into EDTA-containing tubes and centrifuged at 3000 × *g* for 10 min at 4 ℃, and plasma was stored at −80 ℃. Total RNA was extracted from 200 µl plasma samples by using the miRNeasy Serum/Plasma Kit (Qiagen, Hilden, Germany) and cDNA was synthesized using a reverse transcription kit (RiboBio, Guangzhou, China) according to the manufacturer’s instructions. After equal volume dilution of cDNA (20 μl of DNase/RNase-Free Deionized water was added to 20 µl of cDNA), the expression levels of miR-30c-1-3p, miR-432-3p, miR-3154, and miR-379-5p were evaluated by quantitative real-time polymerase chain reaction (qRT-PCR) using specific primers (Supplemental Table 3) and the miDETECT A TrackTM miRNA qRT-PCR Kit (RiboBio) following the manufacturer’s protocol; reactions were performed on a CFX96 Touch™ Real-Time PCR Detection System (Hercules, CA, USA). Each reaction was performed in triplicate, and the relative expression level of miRNAs were calculated based on cycle threshold (Ct) values through formula 2-∆Ct, which ∆Ct value is the Ct value of miRNAs in each patient of URAAA/RAAA group minus the average Ct value of miRNAs in the control group.

### Western blot analysis

Cells and tissues were homogenized using a RIPA buffer (Thermo Fisher Scientific, Glen Burnie, MD) supplemented with protease and phosphatase inhibitors. The total protein was estimated using the BCA protein assay reagent kit (Thermo Fisher Scientific). Briefly, 40 µg total protein was loaded and detected with antibodies against MMP9 (Cell Signaling Technology, Danvers, MA, USA), glyceraldehyde-3-phosphate dehydrogenase (GAPDH) (Cell Signaling Technology).

### Functional annotation of differentially expressed miRNAs

The target genes of differentially expressed miRNAs were predicted using miRWalk, miRanda, RNA22, and Targetscan (http://zmf.umm.uni-heidelberg.de/apps/zmf/mirwalk2/) and analyzed based on GO terms enrichment in three functional categories: molecular function, cellular component, and biological process [26]. KEGG was used for the functional classification of miRNAs according to the relevant biological pathways and for analysis of their relative enrichment.

### Cell culture and transfections

We obtained RAW 264.7 cells and human embryonic kidney HEK293 cell lines from the Chinese Academy of Sciences Cell Bank. Mouse macrophage RAW 264.7 cells and HEK293 cells were maintained in Dulbecco’s Modified Eagle’s Medium (DMEM; Life Technologies Corporation, Carlsbad, CA, USA) supplemented with 10% fetal bovine serum (Life Technologies) at 37 °C in a humidified atmosphere of 5% CO2. RAW 264.7 cells were transfected with a miRNA mimic (100 nM), miRNA inhibitor (100 nM), or their controls (RiboBio, Guangzhou, China) using Lipofectamine RNAiMAX (Invitrogen, Carlsbad, CA, USA) following the manufacturer’s protocol, collected after 48 h, and analyzed for MMP-9 expression by western blotting. HEK293 cells were used for the dual-luciferase reporter assay described below.

### Dual-luciferase reporter assay

PmirGLO Dual-Luciferase miRNA Target Expression plasmids containing the *MMP-9* 3′-UTR or coding sequence were synthesized by GENEWIZ (Suzhou, China) and amplified it in *E. coli*. The plasmids were extracted using the Wizard® Plus SV Minipreps DNA Purification System (Promega, Madison, WI, USA) according to the manufacturer’s instructions.

HEK293 cells were co-transfected with 200 ng of pmirGLO Dual-Luciferase plasmids, and miR-30c-1-3p mimic (100 nM) or mimic control (100 nM) using X-tremeGENE HP DNA Transfection Reagent (Sigma, St. Louis, MO, USA).

Luciferase activity was analyzed by the Dual-Luciferase Reporter Assay System (Promega) following the manufacturer’s protocol. Transfection efficiency was normalized by *Renilla* luciferase activity.

### Biochemical analysis

Serum samples were analyzed for TC (Nanjing Jiancheng Bioengineering Institute, Nanjing, China) and TG (Nanjing Jiancheng Bioengineering Institute) by using the total cholesterol and triglyceride quantification kit according to the manufacturers’ instructions.

### Measurement of MMP-9 plasma concentration

Plasma levels of MMP-9 were determined by enzyme linked immunosorbent assay (ELISA) using a human MMP-9 ELISA kit ( no. ab246539, Abcam, UK) according to the manufacturer’s instructions.

### Statistical analysis

Continuous variables were reported as the mean ± standard deviation (SD) or medians and interquartile ranges (25th or 75th percentiles) and compared by unpaired Student’s *t*-test, one-way ANOVA, or Mann–Whitney *U* test. Categorical variables were expressed as percentages and compared by chi-square test or Fisher’s exact tests. Pearson correlation coefficient (*r*) was used for bivariate normally distributed data. The prediction potential of MMP-9 expression or the maximal aortic diameter was evaluated by ROC curve analysis. The optimal cutoff level was calculated by the Youden index (sensitivity + specificity – 1). GraphPad Prism 8.0 (GraphPad, La Jolla, CA, USA) was used for graphical data presentation. SPSS version 22.0 was used for statistical analysis (IBM, Armonk, New York, NY, USA). The level of significance was set at *P* < 0.05.

## Supplementary Information

Below is the link to the electronic supplementary material.

Supplementary file1 (PDF 78.2 KB)

Supplementary file2 (PDF 58.2 KB)

Supplementary file3 (DOC 75 KB)

## Data Availability

Data and materials will be shared upon request.
