# Correction to: MiR‑30c‑1‑3p targets matrix metalloproteinase 9 involved in the rupture of abdominal aortic aneurysms

**DOI:** 10.1007/s00109-022-02267-3

**Published:** 2022-10-25

**Authors:** Lin Yang, Hong‑Gang Sui, Meng‑Meng Wang, Jia‑Yin Li, Xiao‑Feng He, Jing‑Yuan Li, Xiao‑Zeng Wang

**Affiliations:** Department of Cardiology and Cardiovascular Research, Institute, General Hospital of Northern Theater Command, 83 Wenhua Road, Liaoning, 110016 Shenyang China

**Correction to: Journal of Molecular Medicine (2022) 100:1209–1221** https://doi.org/10.1007/s00109-022-02230-2

The correct assigned affiliation of Xiao‑Zeng Wang is shown in this article.

