# Supplementary material for: MiR-30c-1-3p targets matrix metalloproteinase 9 involved in the rupture of abdominal aortic aneurysms
Source: J Mol Med (Berl). 2022 Jul 15;100(8):1209–21. doi: 10.1007/s00109-022-02230-2 (PMC9329399; doi:10.1007/s00109-022-02230-2)
Supplement: Supplementary file 1 — Supplementary file1 (PDF 78.2 KB) [file 109_2022_2230_MOESM1_ESM.pdf]

Supplemental Figure 1

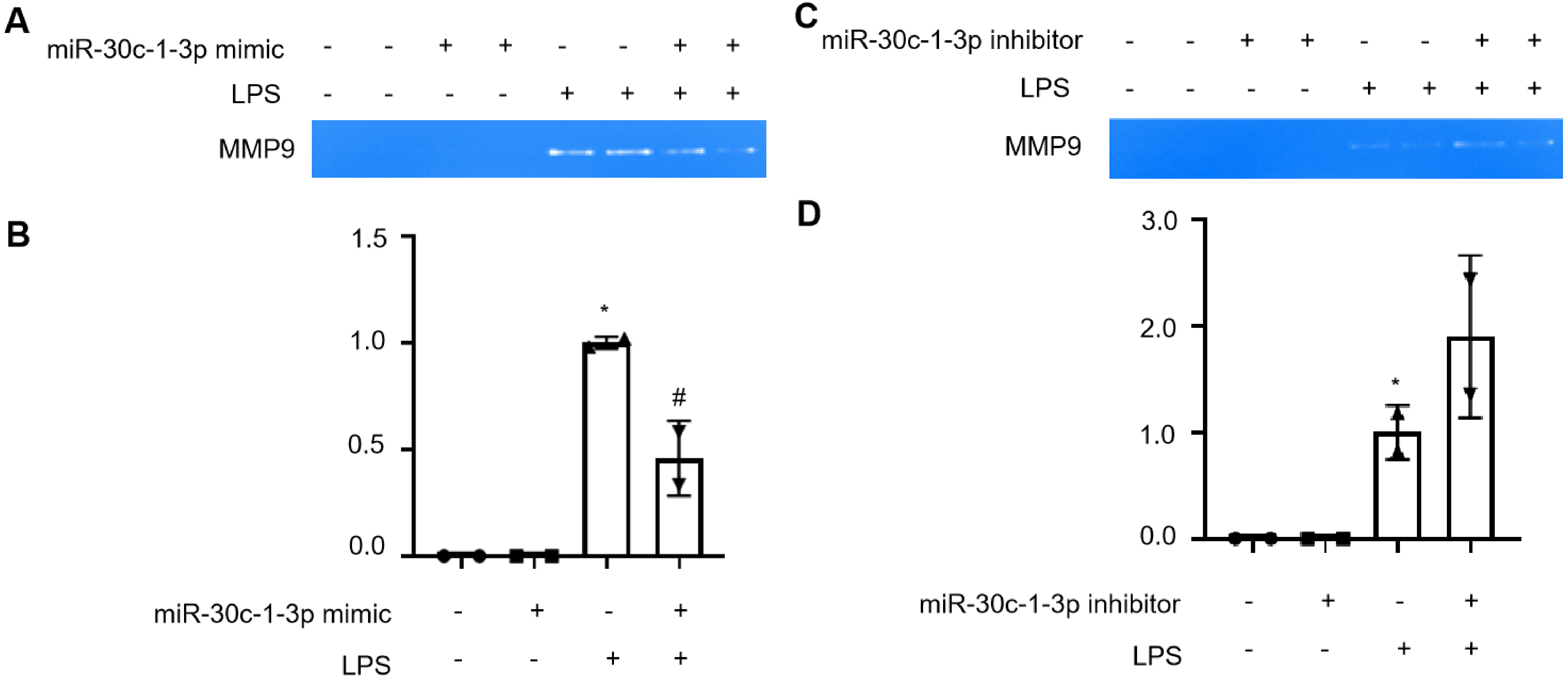

Supplemental Figure 1. The levels of MMP9 in the supernatants of cell cultures were determined via zymography.
A–B. Zymography and quantification of MMP9 in RAW264.7 cells transfected with mimic control or the miR-30c-1-3p mimic with or without lipopolysaccharide (LPS) stimulation. C–D. Zymography and quantification of MMP9 in RAW 264.7, cells transfected with the inhibitor control or miR-30c-1-3p inhibitor with or without LPS stimulation. *P < 0.05, vs. mimic/inhibitor control without LPS; #P < 0.05, vs. mimic control with LPS.
