# Supplementary material for: MiR-30c-1-3p targets matrix metalloproteinase 9 involved in the rupture of abdominal aortic aneurysms
Source: J Mol Med (Berl). 2022 Jul 15;100(8):1209–21. doi: 10.1007/s00109-022-02230-2 (PMC9329399; doi:10.1007/s00109-022-02230-2)
Supplement: Supplementary file 2 — Supplementary file2 (PDF 58.2 KB) [file 109_2022_2230_MOESM2_ESM.pdf]

Supplemental Figure 2

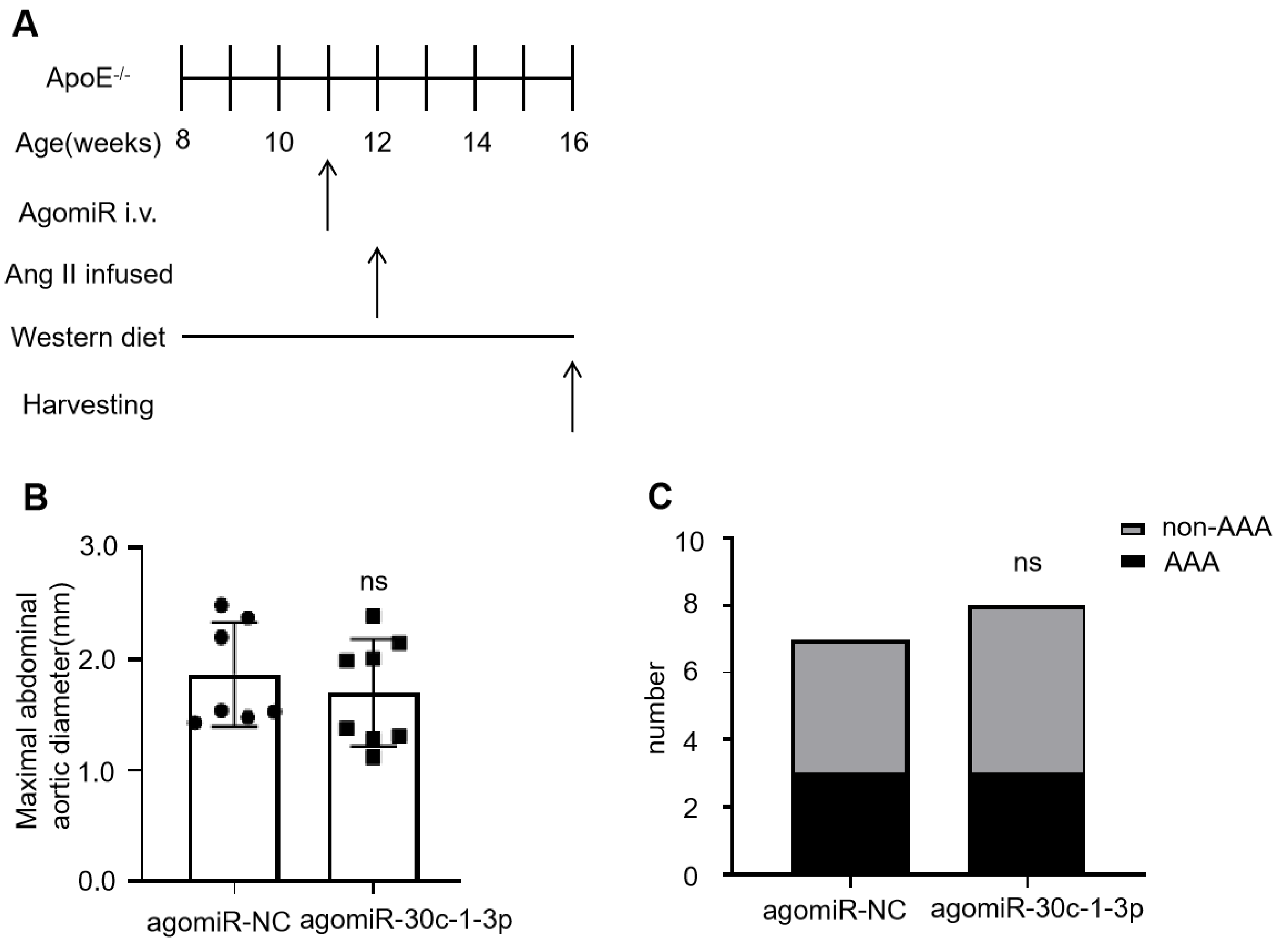

Supplemental Figure 2. Effect of miR-30c-1-3p overexpression on AAA formation rate

(A) Schematic diagram of the mouse model establishment. The maximal abdominal aortic diameter (B) and the formation rate of abdominal aortic aneurysms (C) were calculated in mice with or without agomiR-30c-1-3p treatment (n = 7–8). ns vs. agomiR-NC
