# Supplementary material for: MiR-30c-1-3p targets matrix metalloproteinase 9 involved in the rupture of abdominal aortic aneurysms
Source: J Mol Med (Berl). 2022 Jul 15;100(8):1209–21. doi: 10.1007/s00109-022-02230-2 (PMC9329399; doi:10.1007/s00109-022-02230-2)
Supplement: Supplementary file 3 — Supplementary file3 (DOC 75 KB) [file 109_2022_2230_MOESM3_ESM.doc]

**Supplemental Table 1 Patient Characteristics**

|  | Control(n=19) | URAAA(n=20) | RAAA(n=20) | P值 |
| --- | --- | --- | --- | --- |
| Demographic and clinical data |  |  |  |  |
| Age, y | 58.63±9.59 | 63.55±9.74 | 58.75±10.03 | 0.206 |
| BMI, kg/m^2^ | 25.90±2.88 | 23.99±3.41 | 24.72±6.22 | 0.407 |
| smoking, n (%) | 9(47.37) | 13(65.00) | 16(80.00) | 0.104 |
| alcohol, n (%) | 9(47.37) | 10(50.00) | 10(50.00) | 0.982 |
| Systemic hypertension, n (%) | 10(52.63) | 14(70.00) | 15(75.00) | 0.304 |
| Coronary heart disease, n (%) | 0(0.00) | 4(20.00) | 2(10.00) | 0.118 |
| Cerebral apoplexy, n (%) | 0(0.00) | 2(10.00) | 3(15.00) | 0.233 |
| Diabetes mellitus, n (%) | 3(15.79) | 2(10.00) | 4(20.00) | 0.677 |
| Imaging |  |  |  |  |
| Maximum diameter of aneurysm, cm | -- | 4.78±0.55 | 6.34±1.33 | ＜0.001 |
| Laboratory examinations |  |  |  |  |
| TG, mmol/L | 1.39±0.62 | 1.53±1.03 | 2.08±1.33 | 0.143 |
| TC, mmol/L | 7.08±8.71 | 4.55±0.88 | 12.24±28.94 | 0.406 |
| HDL, mmol/L | 1.11±0.20 | 1.12±0.28 | 1.21±0.81 | 0.831 |
| LDL, mmol/L | 2.24±0.72 | 2.75±0.61 | 3.00±1.10 | 0.027 |
| ALT, U/L | -- | 32.43±53.58 | 132.80±459.63 | 0.364 |
| AST, U/L | -- | 27.10±30.51 | 164.08±582.41 | 0.326 |
| HG, g/L | 147.37±11.14 | 138.53±12.38 | 133.10±22.69 | 0.030 |
| WBC, 10^9^/L | 7.34±2.03 | 7.02±2.33 | 7.94±2.43 | 0.824 |
| PLT, 10^9^/L | 195.92±80.44 | 225.68±51.80 | 196.55±76.69 | 0.340 |
| FPG, mmol/L | 5.89±0.69 | 5.85±1.07 | 6.30±2.46 | 0.665 |
| Medical |  |  |  |  |
| Antiplatelet agents |  |  |  |  |
| Aspirin, n (%) | 19(100.00) | 16(80.00) | 9(45.00) | ＜0.001 |
| Clopidogrel, n (%) | 14(73.68） | 4(20.00) | 4(20.00) | ＜0.001 |
| Ticagrelor, n (%) | 4(21.05) | 0 | 0 | 0.011 |
| Anti-hypertensives |  |  |  |  |
| ACE inhibitors, n (%) | 4(21.05) | 6(30.00) | 11(55.00) | 0.070 |
| ARB inhibitors, n (%) | 3(15.79) | 1(5.00) | 7(35.00) | 0.048 |
| Beta-blockers, n (%) | 15(78.95) | 8(40.00) | 17(85.00) | 0.004 |
| Calcium antagonists, n (%) | 7(36.84) | 9(45.00) | 17(85.00) | 0.005 |
| Diuretic, n (%) | 1(5.26) | 1(5.00) | 5(25.00) | 0.082 |
| Statins, n (%) | 18(94.74) | 13(65.00) | 13(65.00) | 0.050 |
| Anti-coagulants |  |  |  |  |
| Low molecular weight heparin, n (%) | 0 | 7(35.00) | 6(30.00) | 0.018 |
| Fondaparinux sodium, n (%) | 0 | 0 | 1(5.00) | 0.371 |
| Heparin sodium injection, n (%) | 19(100.00) | 6(30.00) | 11(55.00) | ＜0.001 |
| Warfarin, n (%) | 0 | 1(5.00) | 3(15.00) | 0.164 |
| Bivalirudin, n (%) | 0 | 0 | 0 | -- |
| Tirofiban, n (%) | 0 | 0 | 0 | -- |
| Cilostazol, n (%) | 0 | 0 | 0 | -- |
| Rivaroxaban, n (%) | 0 | 0 | 0 | -- |

URAAA, patients with unruptured abdominal aortic aneurysm; RAAA, patients with ruptured abdominal aortic aneurysm; BMI, Body Mass Index; TG, Triglyceride; TC, Total Cholesterol; HDL, High Density Lipoprotein; LDL, Low density lipoprotein; ALT, Alanine Aminotransferase; AST, Aspartate aminotransferase; HG, Hemoglobin; WBC, White Blood Cell; PLT, Platelet Count; FPG, Fasting Plasma Glucose; ACE, Angiotensin-converting Enzyme; ARB, Angiotensin Receptor Blocker. Data are expressed as mean±SD, or number (percentage).
